# Ultrasound biomicroscopy as a vital tool in occult phacomorphic glaucoma

**Published:** 2019

**Authors:** Vasile Potop, Valeria Coviltir, Speranţa Schmitzer, Cătălina Gabriela Corbu, Cătălina Ioana Ionescu, Miruna Gabriela Burcel, Alina Ciocâlteu, Dana Dăscălescu

**Affiliations:** * Ophthalmology Department, “Carol Davila” University of Medicine and Pharmacy, Bucharest, Romania; ** Ophthalmology Department, Clinical Hospital of Ophthalmologic Emergencies, Bucharest, Romania; *** Ophthalmology Department, Oftaclinic Bucharest, Romania

**Keywords:** ultrasound biomicroscopy, glaucoma, lens

## Abstract

Ultrasound is a fundamental tool used in all medical specialties, including ophthalmology. Nowadays, ultrasound biomicroscopy (UBM), a method with higher resolution, allows the investigation of in vivo details of the anterior segment of the eye at microscopic resolution. The examination is especially useful in patients with secondary glaucoma that involves a lens component such as phacomorphic glaucoma and occult phacomorphic glaucoma (OPG). The purpose of this paper was to familiarize audience with UBM and the importance of this investigation in OPG, by presenting a series of cases in which performing an UBM was vital because it provided the information needed in order to safely accomplish a curative surgical treatment that preserved our patients’ visual acuity.

## Introduction

Charles J. Palvin first described UBM over 15 years ago [**1**]. Unlike the conventional ophthalmic ultrasound method that uses 10 MHz, UBM uses ultrasound frequencies up to 50 to 100 MHz range [**1**,**2**]. A high frequency probe is able to penetrate less than a conventional one, but provides better imaging and details [**1**]. The 50 MHz probe is able to penetrate 4 mm, has a resolution of 40 microns and is thought to balance the best depth and resolution [**2**,**3**].

The procedure is very much alike the conventional B scan ultrasonography, but it needs immersion of the transducer in saline solution or 1% methylcellulose in order to be able to give a high-resolution image [**4**]. The signal is then being transmitted to the computer through a signal-processing box [**5**,**4**].

The first structure revealed on UBM is the cornea as a hyperreflective structure. The anterior chamber (AC) depth starting from posterior line of the cornea to the anterior line of the lens could then be measured. Normally, this should be about 2.5-3.0 mm. A flat echogenic area that is the iris, which connects to the ciliary body and the scleral spur, could then be seen. Its insertion and the relationship to the other structures could be investigated. In secondary glaucoma, the iris has a convex appearance due to pupillary block [**3**,**4**]. Lens configuration and diameter should be investigated because it can be useful especially in phacomorphic glaucoma and occult phacomorphic glaucoma. We can also see the sclera that has a high reflectivity.

## Case report

We present a series of 12 cases of patients with OPG in whom UBM played an important role in diagnosis and then in the treatment course and outcome. All patients had a history of hyperopia and primary angle closure glaucoma (PACG) and despite being treated with hypotensive agents and laser iridotomies, they presented with acute painful red eye to the Emergency Room. Clinical examination revealed decreased visual acuity (VA), high IOP and glaucomatous optic neuropathy. After lowering the IOP with systemic hyperosmotic agents and topical medication, we performed an UBM to all these patients. In almost all cases, biometric data showed a small eye with a sagittal diameter under 22 mm, a large IOL and a shallow anterior chamber (AC), or an eye with a sagittal diameter of about 22 mm, but with a small anterior segment – anterior relative microphthalmia associated with a normal lens and shallow AC [**6**,**7**].

**Fig. 1:**
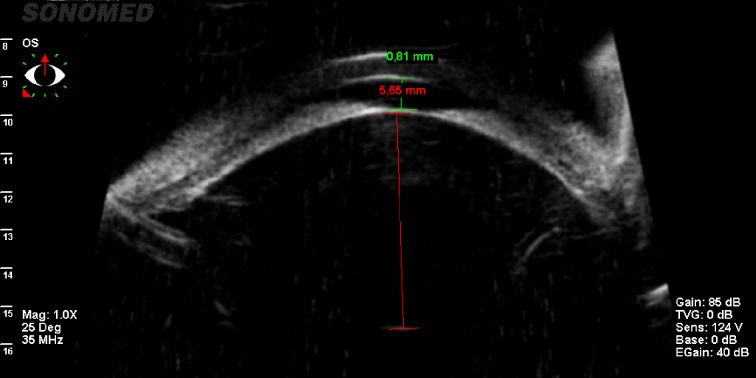
UBM showing a shallow AC and a very large lens in the LE of the patient

We highlighted the case of a 33-year-old patient with a history of LE PACG, laser peripheral iridotomies (LPI) with episodes of eye pain who presented for severe eye pain and visual acuity loss in her LE. Clinical examination revealed best-corrected visual acuity RE 1 Snellen, LE 0,3 Snellen. IOP was 16 mmHg in her RE and 49 mmHg (with maximal topical hypotensive treatment). Fundus examination was normal in RE and revealed advanced optic neuropathy with cup/ disc ratio 0,9 in her LE. UBM revealed a very shallow AC (0,81 mm) and a large lens (5,65 mm sagittal diameter) in her LE (**Fig. 1**).

**Fig. 2:**
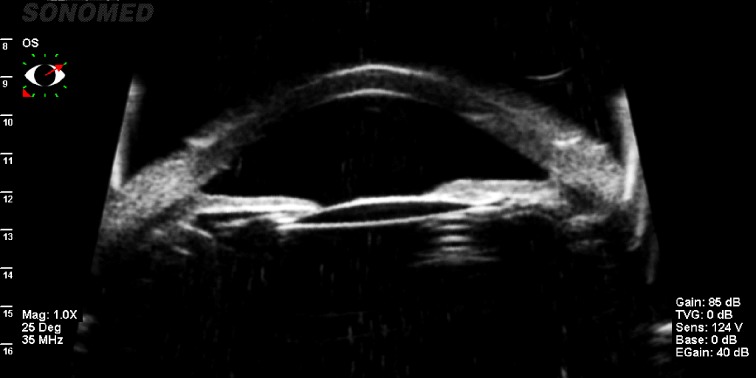
UBM showing the pseudophakic eye with a large AC

**Fig. 3:**
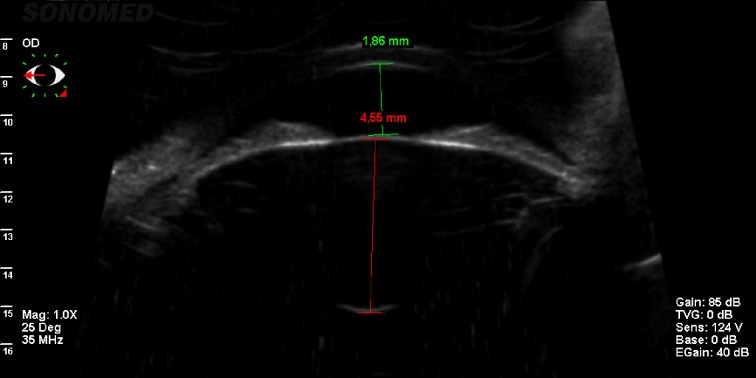
UMB showing a large lens with a shallow AC in the RE of the patient

After lowering the IOP and confirming the phacomorphic component (using UBM), we were able to proceed with a curative treatment: a careful clear lens extraction in her LE. Even if it was a very complicated surgery due to the very shallow AC, large lens, and small pupil, we were able to control the risks knowing exactly the specific anatomy of this eye with the help of the UBM. The postoperative response was positive, with stabilization of the IOP, preserved VA, and normal AC depth (**Fig. 2**). Then, we performed an UBM in her RE that showed a shallow AC (1,86 mm) and a large lens (4,55 mm sagittal diameter) (**Fig. 3**) and we performed LPI with a positive outcome, her IOP remaining stable until now. We monitored the patient closely and in case of an IOP rise in her RE, we were able to perform clear lens extraction.

## Discussions

Glaucoma is globally identified as the leading cause of irreversible blindness [**8**]. The disease is known as a progressive optic neuropathy that determines retinal nerve fiber layer defects and visual field loss [**9**].

Occult phacomorphic glaucoma is a new entity encountered in young individuals. It is a proper phacomorphic glaucoma, with increased lens volume, but the lens is large and clear, unlike the conventional phacomorphic glaucoma that associates intumescent cataract that increases lens volume and determines secondary glaucoma [**7**]. It is frequently encountered in young female patients who associate high IOP and shallow AC that do not respond to medical treatment or LPI. These patients need to be investigated further before proceeding to glaucoma filtrating surgery that could end up in major complications.

UBM is the investigation of choice because it helps evaluate lens dimensions and position. Proving the mechanical role of the lens in the pathophysiology of the disease, we could perform a proper treatment: clear lens extraction, knowing exactly the structure of each eye, the difficulty of the surgery and what possible complications to expect. Performing clear lens extraction in a young individual is a brave and responsible act. However, in OPG the results could be rewarding [**6**].

UBM was able to help investigate further than classic B-scans, providing more detailed images of the anterior segment of the eye, but due to the compromise between depth and resolution it cannot evaluate the posterior segment, so the two methods are complementary used in ophthalmology [**5**,**4**].

Without UBM, the evolution of OPG patients would have been completely different, with multiple surgeries and complications.

## Conclusions

For PACG patients, UBM is a simple noninvasive investigation that could make the difference between refractory primary angle closure glaucoma and an occult phacomorphic glaucoma that was not treated accordingly. It helps evaluate AC, lens-iris relationship and lens diameter. Without it, we might miss the occult phacomorphic component and treat the disease as a regular PACG, neglecting the most important factor that could help our patient preserve his vision.

**Acknowledgment**

All authors had equal participation and contribution to this paper.

**Disclosures**

None.

**Conflicts of interest**

None.
